# Diagnostic implications of lncRNA NORAD in breast cancer

**DOI:** 10.1038/s41598-023-47434-9

**Published:** 2023-11-22

**Authors:** Yaping Zhang, Xiaowei Fan, Jianfeng Hong, Enyu Yang, Cheng Xuan, Hongming Fang, Xianfeng Ding

**Affiliations:** 1 Affliated Xiaoshan Hospital, Hangzhou Normal University, Hangzhou, 311201 China; 2 College of Life Sciences and Medicine, Zhejiang Sci-Tech University, Hangzhou, 310018 China

**Keywords:** Biomarkers, Oncology

## Abstract

This study aimed to assess the expression levels of non-coding RNA activated by DNA damage (NORAD) in the cells, tissues, and serum of breast cancer (BRCA) patients and benign breast nodules and investigate its association with clinicopathological characteristics and prognosis in BRCA. NORAD was analyzed using TCGA-BRCA, GSE77308, Cellminer, and Sangerbox databases, revealing its significance in BRCA prognosis, immune microenvironment, and cell function. Serum samples from 38 BRCA patients, 80 patients with benign breast nodules (50 fibroadenoma and 30 breast adenosis cases), and 42 healthy individuals were collected from Zhejiang Xiaoshan Hospital. NORAD expression was quantified using quantitative reverse transcription PCR (RT-qPCR). Differential NORAD expression between benign and malignant breast nodules and its relationship to clinicopathological characteristics were assessed. NORAD demonstrated elevated expression in BRCA patient serum compared to healthy individuals and those with benign breast nodules (*P* < 0.05). Moreover, its expression correlated with TNM-stage, lymph node metastasis, and luminal classification. These findings highlight the elevated NORAD expression in BRCA patient serum and its correlation with clinicopathological characteristics, providing insights into its potential as a diagnostic biomarker or therapeutic target.

## Introduction

Worldwide, breast cancer (BRCA) is the most frequently diagnosed malignant tumor, accounting for 11.7% of all new malignant tumor diagnoses in 2020, with over 2.26 million new cases reported. Over 680,000 deaths in 2020 from BRCA, comprising 15.5% of total deaths from female malignant tumors. The BRCA mortality rate ranks first among female malignant tumors worldwide; thus, BRCA poses a serious threat to women's health^1^. Despite advancements in surgery, radiotherapy, chemotherapy, targeted therapy, and endocrine therapy, treatment failure still occurs frequently in clinical practice, highlighting the urgent need to unravel the mechanism of BRCA progression^2^.

The development of gene research technology has shed light on the functional roles of long non-coding RNAs (lncRNAs) in various physiological processes and diseases^3^. LncRNAs are a type of non-coding RNA with a transcript length greater than 200 nucleotides found in the nucleus, cytoplasm, and different subcellular organelles of eukaryotic cells. They participate in diverse cell processes such as proliferation, apoptosis, and migration^4^. Many lncRNAs have been linked to cancer biology, carcinogenesis, and metastasis^5^, shedding light on the underlying mechanisms of numerous malignancies, including BRCA^6^. Among them, Non-coding RNA Activated by DNA damage (NORAD) is a lncRNA located on Chr20q11.23 that regulates the stability of the genome by interacting with PUMILIO protein^7^. Abnormal activation of NORAD can disrupt the activity of PUMILIO protein, leading to repression of mitosis, DNA repair, and DNA replication factors, ultimately resulting in chromosome tetraploid^7^.

Scientists have discovered that NORAD is upregulated in response to DNA damage and plays a role in maintaining chromosomal stability in human cells, characterized by high conservation and extensive expression^7,8^. NORAD has been identified as an oncogene in numerous human cancers, where it was found to be frequently upregulated. For example, Tian^9^ reported that NORAD is upregulated in both hepatocellular carcinoma tissues and cells, and it has been revealed that NORAD expression levels were also increased in lung cancer tissues and cells^10^. In esophageal squamous cell carcinoma, NORAD expression is substantially upregulated compared to adjacent normal tissues, and high NORAD expression is associated with tumor size and advanced AJCC staging, according to studies^11^. Multivariate analysis has identified NORAD as an independent predictor of overall survival (OS)^11^. Zhang et al.^12^found that the relative expression level of NORAD in colorectal cancer tissue is significantly upregulated, and its expression level was positively correlated with metastasis and poor prognosis in colorectal cancer patients. Overexpression of NORAD stimulates cell proliferation, migration, and invasion, and it inhibits cell apoptosis through the downregulation of miR-202-5p^12^. Furthermore, it has been reported that knocking out NORAD significantly inhibits the growth and proliferation of breast tumor cells, suggesting its potential oncogenic role^13^.

Currently, research primarily focuses on investigating the expression levels of NORAD in tissue samples to explore its relationship with malignant tumors. However, obtaining tissue specimens for detection can be invasive and traumatic for patients, making it challenging to acquire such samples easily^14^. In contrast, serum samples offer a simpler alternative for assessing NORAD expression. Wang et al.^6^demonstrated significantly higher levels of serum NORAD in patients with colorectal cancer (1.495 ± 1.3024) compared to healthy controls (0.492 ± 0.681) and patients with benign colorectal diseases (1.021 ± 0.975). The ROC curve analysis confirmed the potential of NORAD as a diagnostic biomarker for colorectal cancer. However, there is currently a lack of studies investigating NORAD expression in the peripheral blood of breast cancer (BRCA) patients. In this study, we determined the association of NORAD with prognosis, immune microenvironment, and cell function in BRCA patients using data from the TCGA-BRCA and GSE77308^15^databases. Additionally, we investigated the differences in serum NORAD expression levels between patients with benign and malignant breast nodules. In addition, the correlation between serum NORAD expression and various clinicopathological characteristics of BRCA, such as age, TNM-stage, tumor size, lymph node metastasis, and luminal classification, was investigated.

## Results

### NORAD expression in BRCA cells correlates with cellular functions

**Figure 1:**
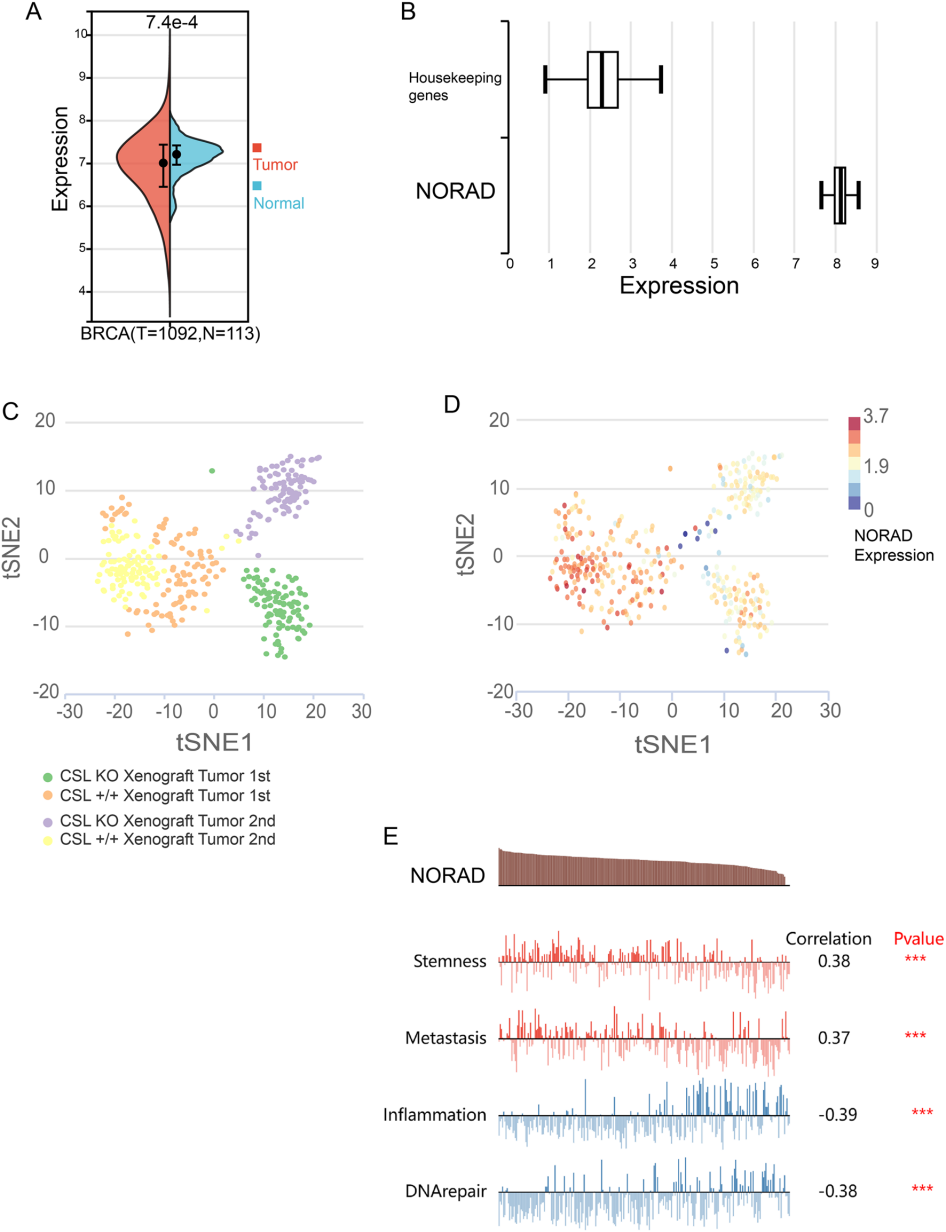
NORAD expression in BRCA tissues and cells. (**A**) Violin plot showing the expression level of NORAD in tumor and non-tumor tissues in the TCGA-BRCA cohort. The expression of NORAD is significantly upregulated in tumor tissues compared to non-tumor tissues. (**B**) Comparison of NORAD expression with housekeeping genes in tumor cells. (**C**) GSE77308 contains 369 cells grouped into four cell groups subjected to Notch activation/inhibition and xenograft experiments. (**D**) Differential expression of NORAD among the cell groups in GSE77308. (**E**) Correlation analysis between NORAD gene expression and cell functions. The top four correlations are depicted, with red indicating a positive correlation and blue indicating a negative correlation. (***P ≤ 0.001).

In the TCGA-BRCA cohort, significant upregulation of NORAD expression was observed in tumor tissues compared to non-tumor tissues, as shown in Fig. 1A. This finding indicates that NORAD may exhibit differential expression in BRCA. Additionally, in the GSE77308 dataset, the expression of NORAD was higher than that of the housekeeping gene, as depicted in Fig. 1B. This further supports the notion that NORAD expression may vary in BRCA.

To investigate the potential role of NORAD in BRCA cells, we examined its expression in CSL knockout (KO) xenograft tumor models. Interestingly, NORAD expression was higher in the CSL wild-type (CSL + / +) xenograft tumor group than in the CSL KO xenograft tumor group, as depicted in Fig. 1C-D. These results suggest that NORAD may serve as a tumor growth-associated marker in tumor cells.

To assess the functional implications of NORAD in tumor cells, we conducted the cellular functional analysis using CancerSEA. Correlation analysis revealed that NORAD expression was positively correlated with stemness and metastasis while negatively correlated with inflammation and DNA repair, as depicted in Fig. 1E. This finding aligns with the previous understanding of NORAD's upregulation in response to DNA damage, as mentioned in the introduction. These results indicate that NORAD exhibits upregulation in tumor tissues and correlates with several important cellular functions in BRCA.

### NORAD expression in BRCA tissues Correlates with Clinicopathological Characteristics

**Figure 2:**
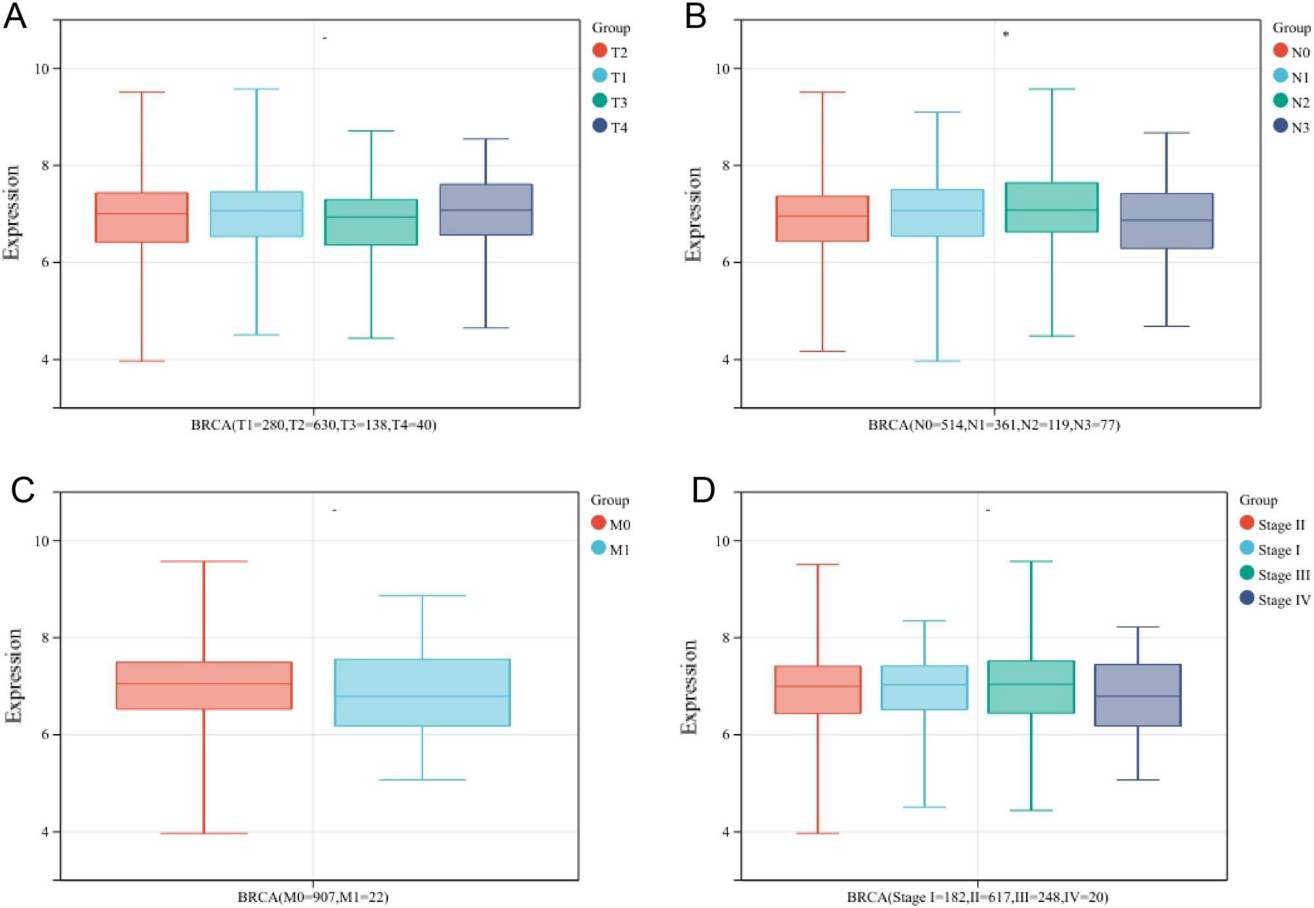
Correlation analysis between NORAD and TNM-stage. (**A**) NORAD expression with the scope and size of the primary tumor (T stage). (**B**) NORAD expression with lymph node dissemination (N stage). (**C**) NORAD expression with metastasis (M stage). (**D**) NORAD expression with TNM-stage. (*P value < 0.05).

To further investigate the correlation between NORAD expression and survival outcomes, we analyzed the NORAD expression in different subgroups based on TNM-stage, OS, disease-specific survival, disease-free interval, and progression-free interval. As shown in Fig. 2 and Supplementary Table S1, we observed differences in NORAD expression among patients with different N stages (N1, N2, N3, N4) with a *P* value < 0.05. In contrast, the invasion-relevant risk score did not correlate significantly with T, M, or TNM-stage.

**Figure 3:**
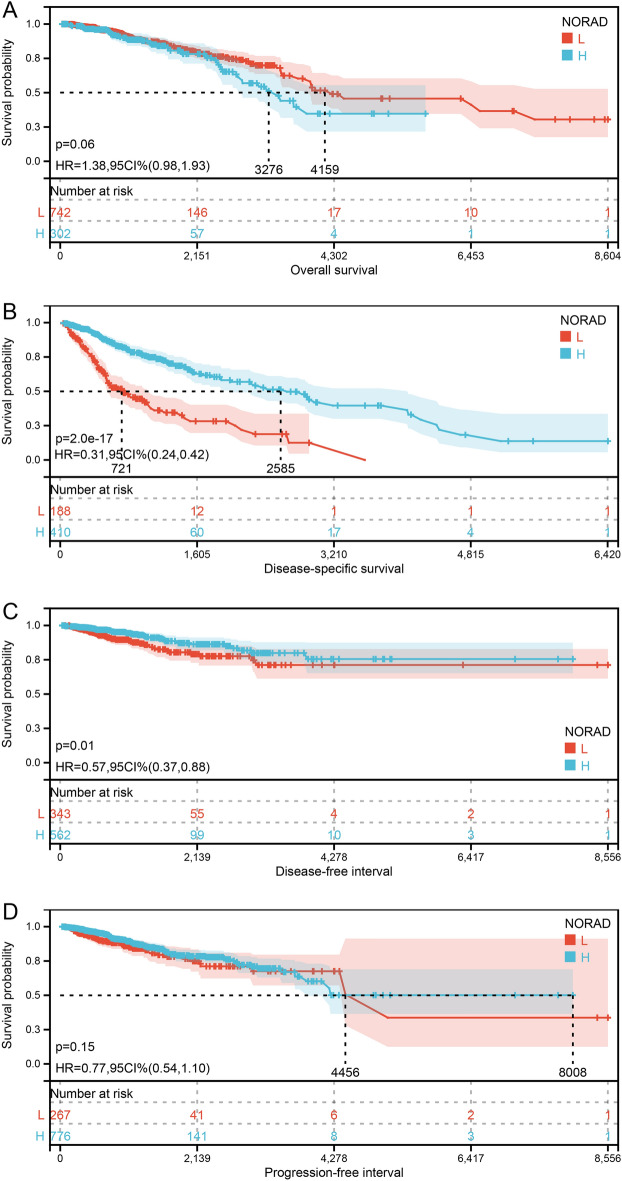
Correlation analysis of NORAD with survival analysis. (**A**) NORAD expression with OS. (**B**) NORAD expression with disease-specific survival. (**C**) NORAD expression with disease-free interval. (**D**) NORAD expression with progression-free interval.

Survival analysis revealed a significant correlation between NORAD expression and disease-specific survival (p-value = 2.0e-17) as well as disease-free interval (p-value = 0.01) (Fig. 3B, C). However, no significant correlation existed between NORAD expression and OS or progression-free interval (*P* value > 0.05) (Fig. 3A, D). These results suggest that NORAD expression may be more associated with the occurrence of BRCA rather than its progression.

**Figure 4:**
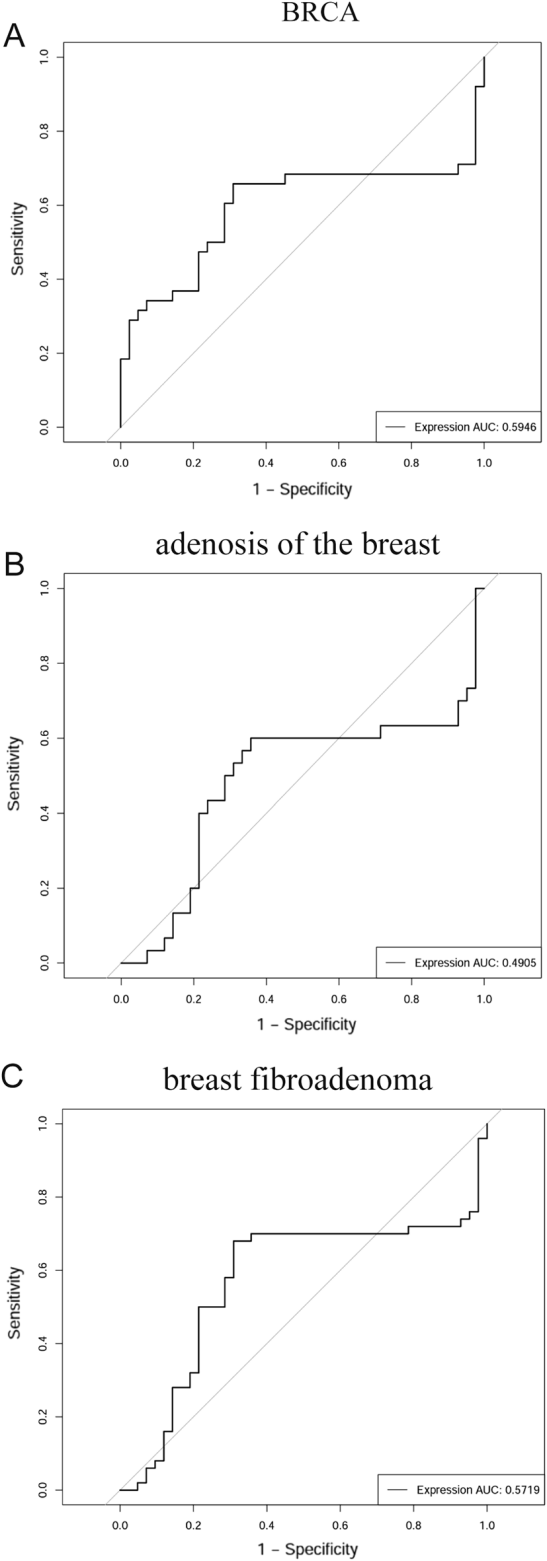
ROC curve analysis of NORAD maped by OmicStudio. (**A**) ROC curve analysis of NORAD in BRCA. (**B**) ROC curve analysis of NORAD in adenosis of the breast. (**C**) ROC curve analysis of NORAD in breast fibroadenoma.

**Figure 5:**
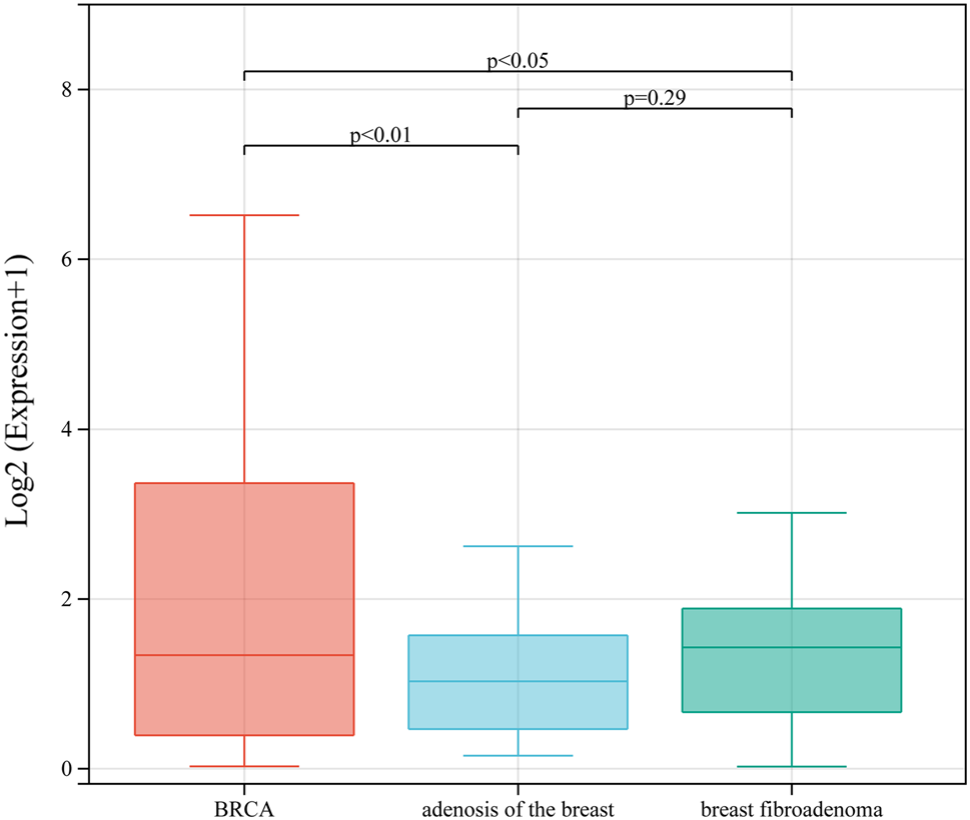
Correlation analysis between NORAD and molecular subtypes.

In order to improve the evaluation of NORAD's diagnostic effectiveness, the receiver operating characteristic curve (ROC) analysis was performed and the related Area Under the Curve (AUC) was calculated. The outcomes, illustrated in Fig. 4A-C, revealed discernible diagnostic potential for NORAD in BRCA and breast fibroadenoma, as evidenced by AUC values of 0.5946 and 0.5719, respectively. However, in the case of breast adenosis, the AUC value was found to be 0.4905. The results (Fig. 5) of this study highlight the diagnostic effectiveness of NORAD in BRCA and breast fibroadenoma, while suggesting a relatively lower ability to distinguish breast adenosis.

### NORAD expression in BRCA tissues correlates with the tumor microenvironment

**Figure 6:**
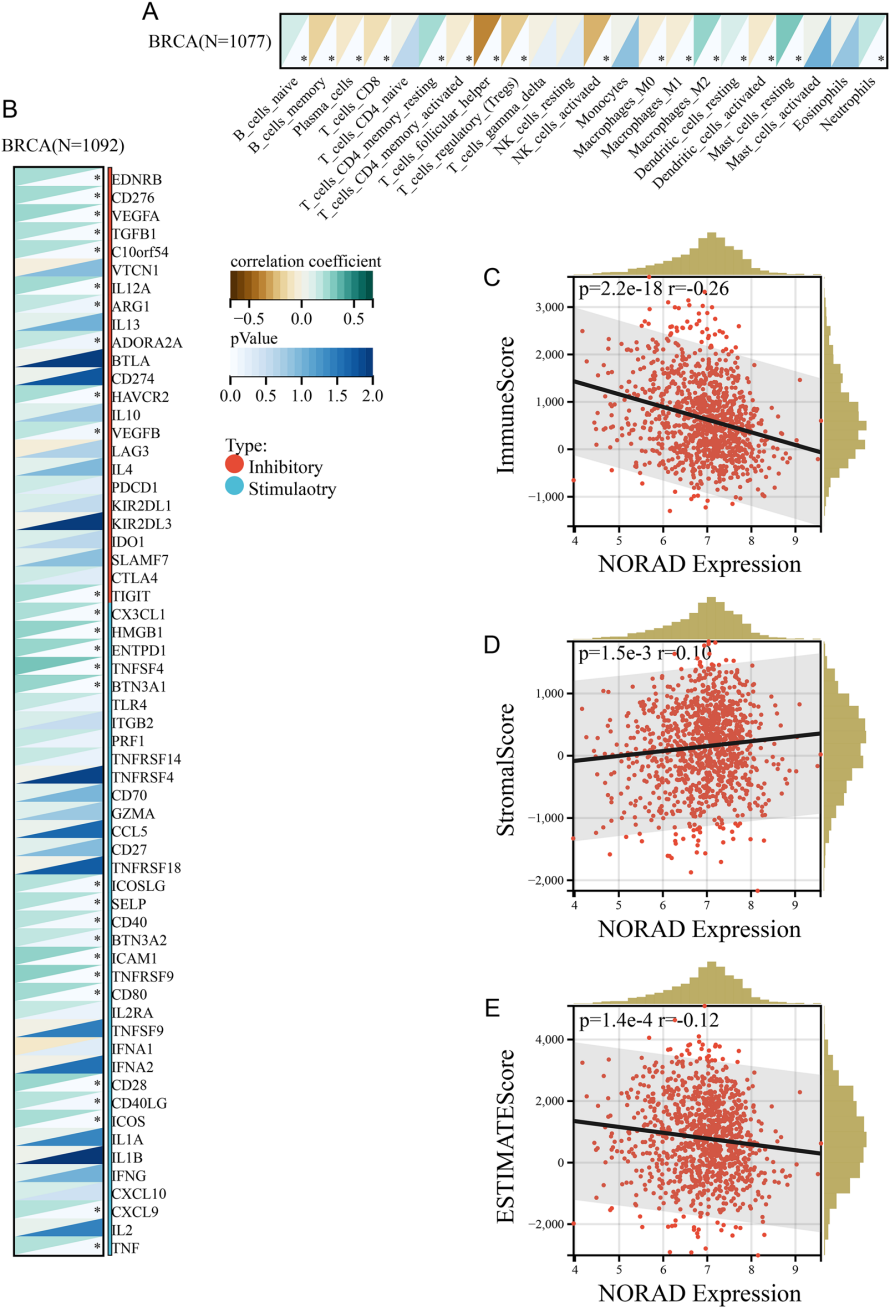
Evaluation of the link between NORAD and the tumor immune microenvironment. (**A**) heatmap of immune infiltration cell analysis (based on CIBERSORT). (**B**) heatmap of immune checkpoint molecules analysis. (**C**) Correlation between the expression of NORAD and the immune score. The column diagram for the horizontal axis displayed the distribution of samples with varying risk scores, whereas the column diagram for the vertical axis displayed the distribution of samples with varying immune scores. (**D**) Correlation between NORAD expression and stromal score. (**E**) Correlation between NORAD expression and ESTIMATE score. (*P value < 0.05).

The CIBERSORT algorithm was used to calculate the scores of immune infiltration cells for each BRCA sample. In Fig. 6A, the scores of various immune cell types were assessed based on an adjusted *P* value threshold of < 0.05. The analysis revealed significant correlations between NORAD expression and the following immune cell types: B cells naive, B cells memory, Plasma cells, T cells CD8, T cells CD4 memory resting, T cells CD4 memory activated, T cells follicular helper, T cells regulatory (Tregs), NK cells activated, Macrophages M0, Macrophages M1, Macrophages M2, Dendritic cells resting, Dendritic cells activated, Mast cells resting, and Neutrophils. This suggests that NORAD expression may play a role in modulating the infiltration of these immune cell populations in BRCA.

We examined the expression of immune checkpoint molecules in the BRCA samples. In Fig. 6B, the expression of 28 immune checkpoint molecules showed significant correlation with NORAD expression based on an adjusted *P* value threshold of < 0.05. This implies a potential involvement of NORAD in regulating immune checkpoint pathways in BRCA.

Finally, the ESTIMATE algorithm and correlation analysis assessed the relationship between NORAD expression and immune scores. Figure 6C-E shows that NORAD expression is significantly negatively correlated with immune score and ESTIMATE score, suggesting higher NORAD expression may be associated with lower immune infiltration and overall immune activity in BRCA. These findings highlight the potential involvement of NORAD in modulating immune cell infiltration, immune checkpoint pathways, and immune activity in BRCA.

### NORAD expression in peripheral blood of BRCA patients is significantly higher than in patients with benign breast nodules

The expression level of NORAD in the serum of BRCA patients (1.71, 0.33, 8.81) was significantly higher than that of healthy individuals (0.70, 0.62, 1.67), breast fibroma patients (1.54, 0.39, 2.68), and breast adenosis patients (1.00, 0.33, 2.14). However, there was no statistically significant difference in serum NORAD expression between healthy individuals and patients with benign breast nodules (*P* > 0.05). Specific values are shown in Table 1.

**Table 1:** The relationship of serum NORAD expression between BRCA, breast fibroadenoma, adenosis of the breast, and healthy individuals.

Groups	Cases	NORAD	Z value	*P* value
			− 2.64	0.008
BRCA	38	1.71 (0.33, 8.81)		
Healthy individuals	42	0.70 (0.62, 1.67)		
			− 2.01	0.044
BRCA	38	1.71 (0.33, 8.81)		
Breast fibroadenoma	50	1.54 (0.39, 2.68)		
			− 2.59	0.010
BRCA	38	1.71 (0.33, 8.81)		
Adenosis of the breast	30	1.00 (0.33, 2.14)		
			− 1.68	0.093
Breast fibroadenoma	50	1.54 (0.39, 2.68)		
Healthy individuals	42	0.70 (0.62, 1.67)		
			− 0.23	0.816
Adenosis of the breast	30	1.00 (0.33, 2.14)		
Healthy individuals	42	0.70 (0.62, 1.67)		
			− 1.50	0.134
Breast fibroadenoma	50	1.54 (0.39, 2.68)		
Adenosis of the breast	30	1.00 (0.33, 2.14)		

### The relationship between serum NORAD expression and clinicopathological characteristics of BRCA

The expression of NORAD in the serum of BRCA patients with TNM ≥ IIb was significantly higher (*P* = 0.004) than in patients with TNM < IIb. Additionally, the expression of NORAD in BRCA patients with lymph node stage N1 + N2 was significantly higher compared to N0 (*P* = 0.036). Moreover, the expression of NORAD in luminal type D was lower than in luminal type A, B, and C (*P* < 0.05). However, there was no correlation between the expression level of NORAD in the peripheral blood of BRCA patients and age, T stage, and tumor size (*P* ≥ 0.05). Refer to Table 2 for detailed information.

**Table 2:** The relationship between serum NORAD expression and clinicopathological characteristics of BRCA.

Clinicopathological	Total (n = 38)	NORAD expression M(P25, P75)	Z value	*P* value
Age(years)			− 1.96	0.05
≥ 50	24	1.85(0.35, 1.85)		
< 50	14	0.96(0.29, 5.17)		
TNM-stage			− 2.88	0.004
0 + I + IIa	28	1.42(0.30, 5.67)		
IIb + III + IV	10	5.32(1.24, 38.45)		
Tumor size			− 0.089	0.929
≥ 2 cm	21	1.58(0.34, 9.17)		
< 2 cm	17	2.09(0.32, 7.86)		
T stage			− 0.26	0.797
Tis + T1	18	1.86(0.34, 7.62)		
T2 + T3	20	1.70(0.33, 9.26)		
N stage			− 2.10	0.036
N0	26	1.53(0.32, 5.37)		
N1 + 2	12	3.64(0.64, 37.58)		
Luminal subtypes			− 2.83	0.005
A	17	2.19(0.64, 10.06)		
D	2	0.38(0.02. 0.88)		
Luminal subtypes			− 2.09	0.037
B	13	1.54(0.28, 4.26)		
D	2	0.38(0.02. 0.88)		
Luminal subtypes			− 2.56	0.011
C	4	5.64(0.86, 11.48)		
D	2	0.38(0.02. 0.88)		

### Relationship between NORAD levels and drug sensitivity

**Figure 7:**
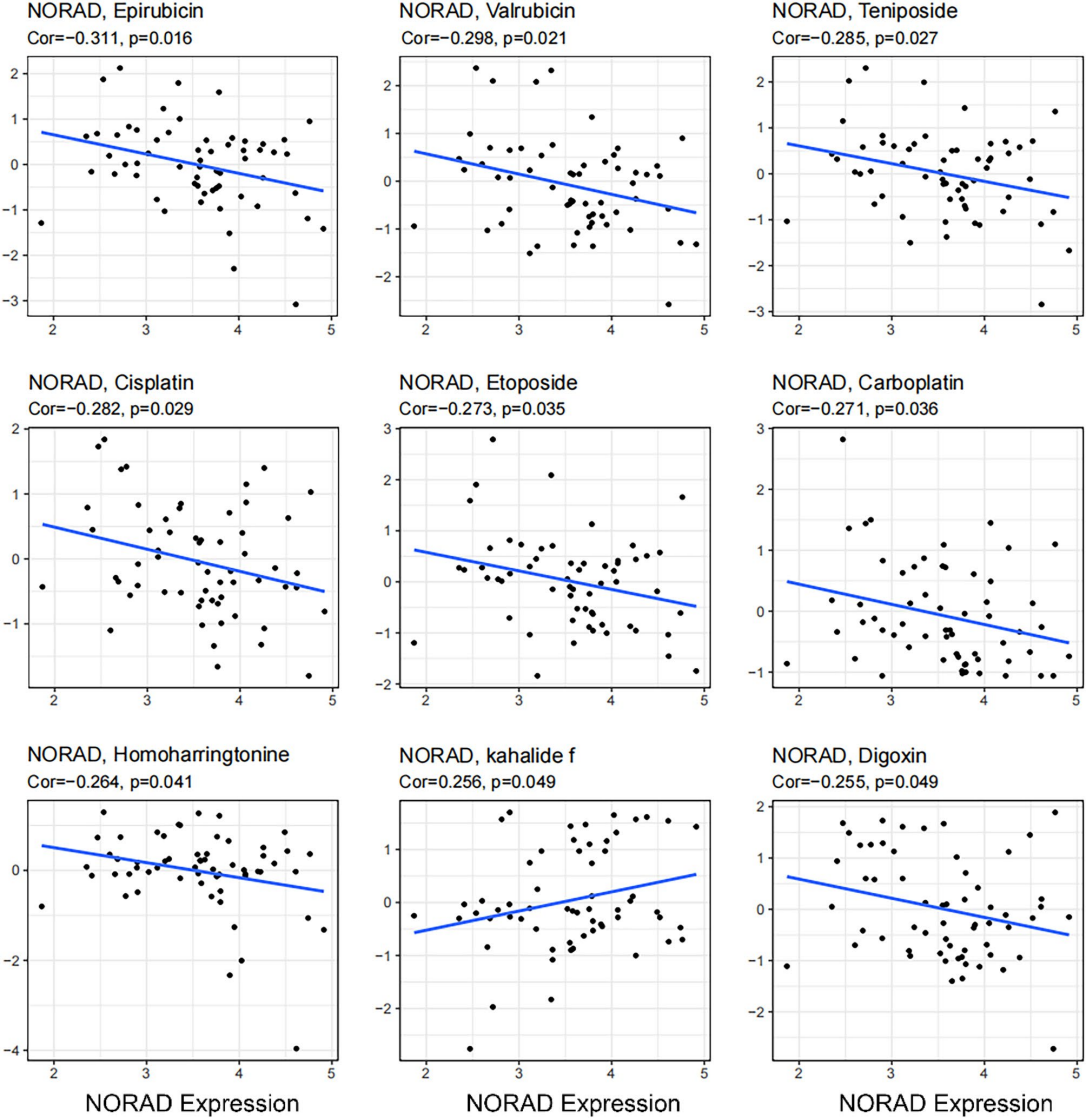
Polt of the association between NORAD levels and drug sensitivity.

We evaluated the correlation between NORAD levels and drug sensitivity using CellMiner data. Figure 7 displays the 9 drugs that exhibit the most robust correlations. The drug sensitivity of Epirubicin, Valrubicin, Teniposide, Cisplatin, Etoposide, Carboplatin, Homoharringtonine, Digoxin, Nelfinavir exhibited a negative correlation with NORAD levels, while a positive association was observed with the sensitivity of kahalide f. The results of this study indicate that the measurement of NORAD levels may serve as a viable approach to selecting anticancer drugs.

### Functional enrichment analysis of NORAD

**Figure 8:**
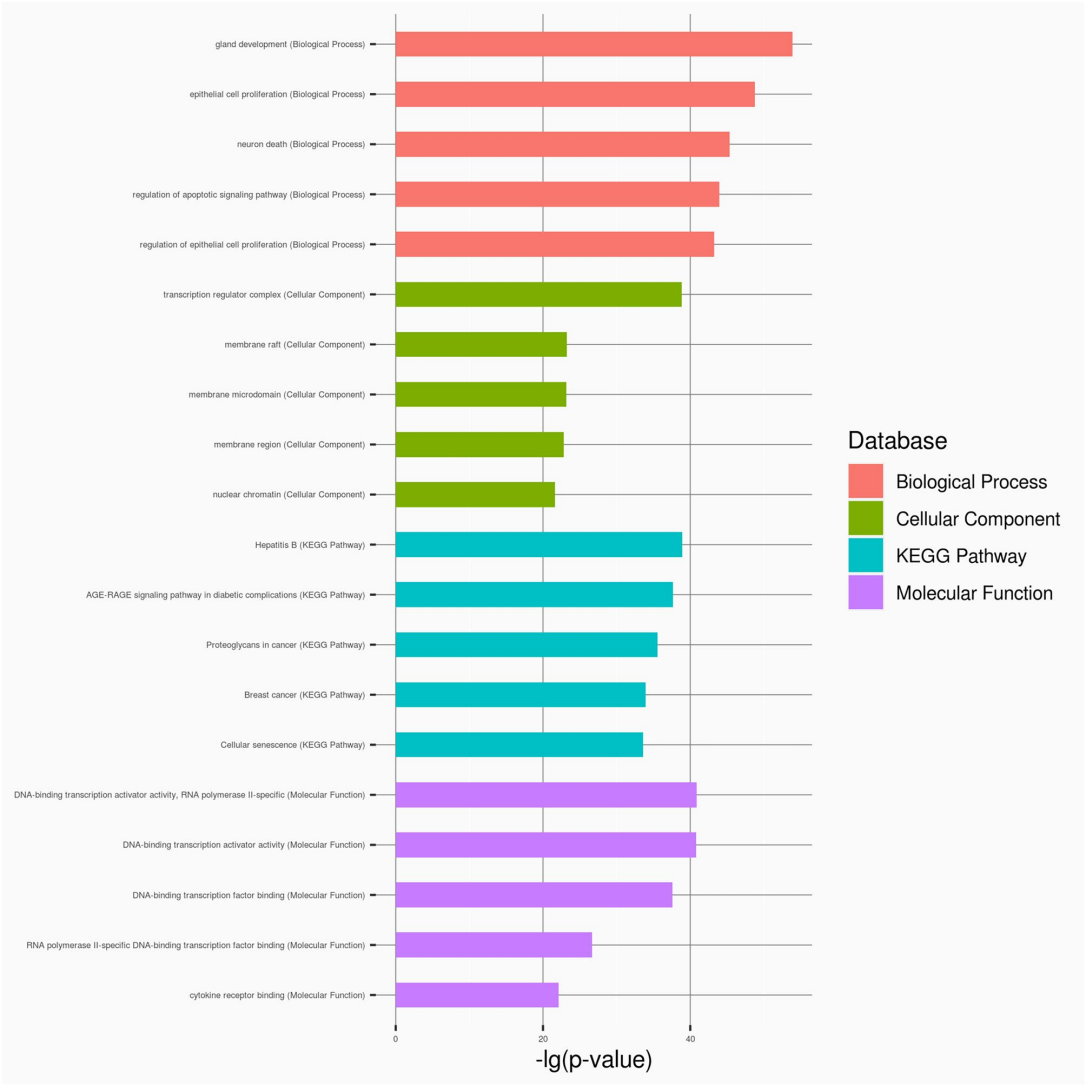
The bar plot of GO enrichment analysis and KEGG pathway analysis of NORAD was analyzed using RNAenrich.

The results (Fig. 8) of the gene ontology (GO) analysis revealed that NORAD plays a prominent role in various biological processes, including gland development, epithelial cell proliferation, neuron death, regulation of apoptotic signalling pathway and regulation of epithelial cell proliferation. Furthermore, NORAD was found to be significantly enriched in cellular components such as the transcription regulator complex, membrane raft, membrane microdomain, membrane region and nuclear chromatin. The NORAD gene has a notable enrichment in various molecular functions, including DNA-binding transcription activator activity specific to RNA polymerase II, DNA-binding transcription factor binding and RNA polymerase II-specific DNA-binding transcription factor binding. According to KEGG analysis, NORAD is predominantly enriched in hepatitis B, the RAGE signalling pathway in diabetes complications, Proteoglycans in cancer, breast cancer and cellular senescence. These findings collectively highlight the multifaceted involvement of NORAD in crucial biological processes and molecular functions, shedding light on its potential significance in various physiological contexts.

## Conclusions

In conclusion, our study revealed a significant correlation between NORAD and prognosis, immune microenvironment, and cell function in BRCA. We observed elevated NORAD expression in the peripheral blood of BRCA patients compared to healthy individuals and those with benign breast nodules. This elevated expression was associated with late TNM-stage and positive lymph node metastasis, suggesting its potential as a diagnostic and differentiating marker for benign and malignant breast nodules. NORAD holds promise as a biomarker for BRCA diagnosis and monitoring. However, further research with larger cohorts is necessary to validate these findings and elucidate the underlying mechanisms of NORAD in BRCA pathogenesis.

## Discussion

BRCA is a complex, heterogeneous disease with many genetic variations and intrinsic subtypes, including luminal subtypes, which arise from luminal epithelial cells of mammary glands, and non-luminal subtypes that are derived from myoepithelial and basal cells of mammary glands^16,17^. The precise etiology of most BRCAs is unknown due to the complexity and diversity of these subtypes^18,19^. Previous studies have shown that the lncRNA NORAD is upregulated in BRCA tissue, promotes the proliferation, invasion, and migration of BRCA cells, and is associated with poor prognosis^20^. Consistent with previous findings, our study revealed that the expression level of NORAD in peripheral blood was significantly higher in BRCA patients compared to healthy individuals and patients with benign breast nodules, suggesting that the expression level of NORAD in peripheral blood may be a predictive value for BRCA.

Some studies have reported that high expression of NORAD is associated with high histological grade, larger tumor size, and advanced clinical stage of BRCA^21^. Wang et al.^22^ conducted a meta-analysis and found a correlation between the high expression of NORAD in tumor tissue and low differentiation, positive lymph node metastasis, and larger tumor volume. Our study also observed a correlation between high expression of NORAD in the serum of BRCA patients and late TNM-stage and positive lymph node metastasis, which is consistent with the previous findings from tissue samples. These results also suggest that NORAD may play a role in the development, carcinogenesis, and progression of BRCA, and peripheral blood NORAD detection may have the potential to replace tissue NORAD detection. Although our study showed a correlation between NORAD expression and luminal subtypes, it is important to note that the sample size of luminal D subtype was small in this study, which may introduce bias. Further research with larger cohorts is needed to validate this finding and explore the clinical implications of NORAD in different subtypes of BRCA.

Our survival analysis revealed a strong relationship between NORAD expression and DSS as well as DFI, and ROC curve studies showed significant potential in the diagnosis of breast fibroadenoma and BRCA. These results strongly suggest that NORAD has the ability to diagnose breast disease and that it may be an important biomarker for prognostic assessment and patient management. More precisely, our findings suggest that shorter DSS and DFI in BRCA patients are associated with increased NORAD expression, suggesting that NORAD may be involved in tumor growth, metastasis and resistance to treatment in patients with BRCA malignancies. Although breast fibroadenoma is generally considered a benign disease, the ROC curve of NORAD in BRCA and breast fibroadenoma shows high diagnostic potential. This is especially important because some fibroadenoma patients may experience worsening disease, highlighting the role of NORAD in early diagnosis and prediction. The assessment of NORAD levels concerning drug sensitivity highlights strong correlations with nine drugs, implying NORAD may serve as a valuable factor in the process of selecting anticancer drugs. The importance of NORAD in pathways linked to illnesses such as cellular senescence and breast cancer is shown by KEGG analysis. In essence, our findings shed light on the nuanced involvement of NORAD in crucial physiological contexts.

In the past, NORAD-related research has primarily focused on detection from tissue samples, which can be difficult to obtain due to the invasive nature of the acquisition procedure. In contrast, our study used RT-qPCR to detect NORAD levels in peripheral blood, which is convenient, fast, and well-tolerated by patients. Although there are limitations in peripheral blood RNA detection in terms of stability relative to tissue RNA detection, the simplicity and ease of peripheral blood testing make it a promising approach for clinical practice. Additional limitations of our study include a relatively small number of BRCA specimens and the need for further validation with a larger sample size.

## Methods

### Data source

The TCGA-BRCA cohort, consisting of 1092 tumor tissues and 113 adjacent non-tumor tissues, was obtained from the UCSC database^23^ (https://xenabrowser.net/). The data were uniformly normalized. Specifically, NORAD (LINC00657) expression data were extracted from each sample and subjected to log2(x + 0.001) transformation.

In addition, we acquired the GSE77308 dataset, which comprises patient-derived xenograft BRCA samples that underwent single-cell RNA-seq. To further explore the data, we utilized the CancerSEA^24^ (http://biocc.hrbmu.edu.cn/CancerSEA).

### Sample collection and clinical information

Peripheral blood was collected from 38 patients with BRCA, 80 patients with benign breast nodules (including 50 cases of breast fibroadenoma, 30 cases of adenosis of the breasts), and 42 healthy individuals at the Zhejiang Xiaoshan Hospital from February 2021 to September 2022. Both benign and malignant breast nodules were confirmed by cytopathology or histopathology. The age of the participants ranged from 15 to 82 years old. In the BRCA group, 32 patients had relatively complete postoperative pathological data; 14 patients were younger than and 24 patients were older than 50 years old. TNM-stage included 6 cases in stage 0, 8 cases in stage I, 14 cases in stage IIa, 5 cases in stage IIb, 2 cases in stage IIIa, and 3 cases in stage IV. 3 cases had masses > 5 cm, and 35 cases had masses ≤ 5cm. Lymph node staging revealed the following: N0 in 26 cases, N1 in 10 cases, and N2 in 2 cases; 3 cases had distant metastasis and 35 cases had no distant metastasis. 2 cases had nerve invasion, while 30 cases had no nerve invasion. Moreover, 5 cases had vascular metastasis and 27 cases had no vascular metastasis. 17 cases were of Luminal A type, 13 cases of B type, 4 cases of C type, and 2 cases of D type. Ultrasound staging included 25 cases in stage IV, 12 cases in stage V, and 1 case in stage VI. The collected serum samples were frozen in liquid nitrogen and stored at -80℃.

The study protocol was approved by the Medical Ethics Comittee of Zhejiang Xiaoshan Hospital (approval number: 2021–2019), which waived the requirement for informed consent due to the retrospective design of this study. This study was conducted according to the Declaration of Helsinki^25^.

### TNM-stage and clinicopathological characteristics analysis

TNM-stage and survival analysis was performed using R software (version 3.6.4). Firstly, the differential expression of the NORAD in different clinical stage samples within BRCA was calculated. To assess the significance of pairwise differences, a non-paired Student’s t-test was utilized. For comparing differences among multiple groups, analysis of variance (ANOVA) was used.

A quality TCGA-BRCA prognosis dataset obtained from a previous study and Sangerbox database^26,27^ was used. Samples having a follow-up period of less than 30 days were eliminated. The optimal cutoff value for NORAD was determined using the R package ‘maxstat’ (version: 0.7–25). Minimum subgroup sample size greater than 25% and maximum subgroup sample size less than 75% were used to determine the cutoff value. Consequently, this cutoff value separated patients into groups with high and low expression. The prognostic differences between the two groups were determined using the ‘survfit’ function from the ‘survival’ R package. The log-rank test was utilized to evaluate the significance of prognostic differences between groups. Furthermore, in order to evaluate the performance of clinical data, we used the OmicStudio tools^28^ (https://www.omicstudio.cn/tool/58) to perform Receiver operating characteristic (ROC curves).

### Tumor immunity analysis

The immune score and stromal score were computed using the R 'estimate' package (version 1.0.13)^29^ for the immune score and stromal score, respectively. For immune-infiltration analysis, the R package ‘IOBR’ (version 0.99.9)^30^ was utilized.To determine the significant correlations between gene expression and tumor immunity in BRCA, we calculated Pearson’s correlation coefficient utilizing the 'corr.test' function from the ‘psych’ package (version 2.1.6).

### RT-qPCR

Total RNA was isolated from serum with TRIzol reagent (AG RNAex Pro Reagent, China) following protocol and reverse transcript into cDNA using HiScript II 1st Strand cDNA Synthesis Kit (Vazyme). Quantitative reverse transcription PCR (RT-qPCR) was performed to determine the expression of NORAD using SYBR Green Pro Taq HS premixed qPCR kit II (with ROX) (AG, China) on the ABI 7500 System (Thermo Fisher Scientific). The primer for NORAD is as follows: forward, 5′-GGAAGAGGGAGAAGAGGA-3′, reverse, 5′-CACAATGAACACAGGCAC-3′. The primer for GAPDH follows: forward, 5′-ACAACTTTGGTATCGTGGAAGG-3′, reverse, 5'- GCCATCACGCCACAGTTTC-3′. The GAPDH was used as endogenous controls for NORAD, and the experiment was repeated three times.

### Drug sensitivity evaluation

We assessed the influence of NORAD levels on drug sensitivity. Drug sensitivity and gene expression data were retrieved from the CellMiner^31^ database, a comprehensive repository that gathers, processes, and consolidates molecular data on NCI-60 and other cancer cells.

### GO enrichment analysis and KEGG pathway analysis

Gene Ontology (GO) enrichment analysis and Kyoto Encyclopedia of Genes and Genomes (KEGG) pathway analysis are essential to gain insights into the biological functions and pathways associated^32–34^ with NORAD. In this context, we utilize RNAenrich^35^ ( https://idrblab.org/rnaenr/), a tool designed for the enrichment analysis of functional annotations for NORAD.

### Statistical analysis

Statistical analyses were performed using SPSS 22.0. Non-normal distribution data are presented as median (M) and quartile interval (P25, P75), and non-parametric tests were used for inter-group comparisons. A *P* value less than 0.05 was considered statistically significant.

### Supplementary Information

Supplementary Table 1.

Supplementary Table 2.

## Data Availability

The datasets used and/or analyzed during the current study are available from the corresponding author on reasonable request.
